# Etiology and Pathology of Uterine Displacements1Read before the Buffalo Academy of Medicine, December 22, 1896.

**Published:** 1897-04

**Authors:** M. A. Crockett

**Affiliations:** Gynecologist to the Erie County Hospital, clinical lecturer in gynecology University of Buffalo


### ETIOLOGY AND PATHOLOGY OF UTERINE DISPLACEMENTS.[1]

By M. A. CROCKETT, M. D.,

Gynecologist to the Erie County Hospital, clinical lecturer in gynecology University of Buffalo.

AS AN introduction to the discussion of this subject, it is proper, briefly, to review the anatomical relations of the uterus as constituting its mechanism of support. The apex of this organ is inserted into the pelvic floor. The anterior vaginal wall and utero-sacral ligaments form an elastic beam stretching across the pelvis and permitting a certain degree of up-and-down movement to the cervix. The posterior vaginal wall is in contact with the anterior, hence excessive descent of the elastic beam is met by the perineum. Experiments show that as long as this beam is not subjected to abnormal weight or strain, it can support the uterus unaided.

1. Read before the Buffalo Academy of Medicine, December 22, 1896.

The utero-sacral are the only uterine ligaments which remain constantly taut, thus insuring the pulling back of the cervix and the tipping forward of the fundus ; in this position the posterior wall of the uterus receives a downward thrust from intra-abdominal pressure.

Both the broad and round ligaments are quite slack, but the contents of the pelvis bear down upon these structures so that continuous traction is exerted by them. As the uterus moves backward or sideways the tension of these ligaments becomes progressively greater until it balances the displacing force.

There has been much discussion concerning the normal position of the uterus, but, as we can readily see, there are a great many normal positions, each of which represents an adjustment to the forces active at that particular time. The posture of the woman, the degree of bladder distension and the amount of bowel contents produce incessant variation of intra-pelvic pressure requiring constant change in the uterine position. Operations which permanently fix the uterus in any one position are faulty from a physiological standpoint ; they prevent, or hinder, that delicate adjustment to varying relations which is the prominent characteristic of a normal uterus.

The axis of the superior strait passes through the center of what may be designated the physiological area of uterine movement. That is, if we swing the fundus around the pelvis in the largest circle permitted by the uterine attachments, that circle will represent the base of a cone whose apex is the cervix fastened to the pelvic floor ; the axis of this cone will coincide with that of the pelvic inlet. During labor we know how important it is for the uterine forces to act in the right direction ; nature, while permitting a certain degree of movement, has arranged for the fundus to be at the right place in the right time.

From a pathological point of view, a uterus is displaced whenever it permanently takes up a position outside of the limits of this physiological area ; for in such positions the normal intra-pelvic force, instead of replacing, still further displaces the organ. This is particularly the case when the fundus has passed backward beyond the line of the perpendicular, so that all downward pressure falls upon the anterior uterine wall, both preventing its forward return and gradually overcoming the tension of the round ligaments.

Some writers require the presence of symptoms before classing a displacement as pathological, but the development of symptoms will depend upon the powers of resistance in the organism ; their presence or absence, while affecting the question of treatment, has nothing to do with that of classification. Retroversions, as well as refractive errors and cardiac disease, have been discovered by accident; they represent conditions which, sooner or later, will produce symptoms ; their treatment comes under the head of preventive medicine.

For normal working of the mechanism of uterine support two requirements must be fulfilled : these are normal tissues and normal forces. Thus, the causes of uterine displacements may be divided under two heads : the first includes all those conditions which impair the integrity or tonicity of the supporting structures; the second, all abnormalities in the amount or direction of the forces acting upon the uterus. Of course, the most serious displacements are those brought about by causes which act under both these heads.

It is of vital importance for the specialist to bear in mind those conditions which produce deficient tone in the supporting structures, for many of these conditions are general and not local. The best gynecologist is the one who knows when to leave the pelvic organs alone and turn his attention to the body at large. The faults of dress, diet and education, every disease or hygienic error which results in lowered vitality, may play a part in producing such a degree of structural weakness that the uterus is unable to stand up under the stress of daily life. From some of these general causes, or from lack of development, that flabby condition of the uterine walls may result which permits the various flexions to occur. In treating such cases, the nervous, nutritive and circulatory apparatus must receive special attention.

Subinvolution following abortion or labor gives rise to conditions falling under both the divisions mentioned above ; the congestion of the pelvis lowers the tone of the pelvic floor and, at the same time, gives a heavier uterus to support. As a consequence, the elastic supporting beam sags and comes down upon the perineum for a resting-place. If the strength of this body is impaired by rupture, or otherwise, a still further descent of the uterus occurs. In the mechanism of labor, whatever comes down first goes to the front ; as the uterus gets lower, the cervix, like the occiput, turns forward and a retroversion is produced. This position brings the uterine axis into the line of the inferior strait, so that the womb may easily be forced down hill into the outer world. In treating such cases it is important to bear in mind that the descent is the primary factor ; retroversion is a result, not a cause.

There is another class of cases which depends, primarily, upon relaxation of the uterine walls and ligaments. Some sudden shock throws the fundus backward and, if the utero-sacral ligaments hold, a flexion is produced. Very often the retractores stretch, the cervix comes forward and we have a version combined with an amount of descent in proportion to the degree of relaxation in the utero-sacral ligaments. If the perineum is strong, descent is prevented, and we have to deal only with a retroversion.

Under the head of abnormal forces we may class those cases in which the uterus is subjected to heavy pressure—as instances we may mention, the increase in intra-abdominal pressure from heavy lifting, straining from constipation, growing tumors, waist constriction, and the like. The uterine supports are adjusted for certain weights ; when overloading occurs they soon lose their tonicity and introduce another pathological element into the case.

It is needless to give a detailed account of all the causes which bring about uterine displacements ; by bearing in mind the general classification the mechanism is easily understood. The strength of the uterine ligaments depends chiefly upon the presence of muscular fibers prolonged from the wall of the uterus. This being so, it follows that metritis not only impairs the tonicity of the womb itself, but also that of its supports. The majority of misplaced uteri are subjected to disturbances of circulation leading to venous engorgement. Such congestion aggravates an old metritis or produces the condition favorable for its development, and, in time, such atrophy of the ligaments may occur as to render them useless for future support ; such a condition, when present, has an important bearing in the selection of operative measures.

The symptoms of a displacement may be those of a metritis alone, but usually something more is added. There is a demand for the expenditure of nervous energy in every effort made by the body tissues to perform their functions ; a displacement produces an element of increased resistance, and the nervous system, sooner or later, fails to meet the demand and various disturbances arise. There is nothing more difficult than to decide what part is played by a displaced uterus in producing symptoms referred to distant parts of the body. It is best to make very guarded promises as to results of treatment ; act upon the general principle of setting right whatever is manifestly wrong. To see a headache disappear merely by replacing a retroverted uterus has, on several occasions, been a source of great surprise to the writer; more frequently has he seen no benefit whatever from such treatment. There seems to be no way to know beforehand which one of several pathological conditions is responsible for the symptoms; the only safe rule is to treat them all.

The prolapsing of ovaries, pressure upon the rectum and pelvic nerves are conditions so easy to understand that comment is unnecessary.

There is a class of physicians who greatly over-estimate the effects of displacements, but there are also those who overlook the pelvis entirely. The best rule is to avoid both these extremes and rest upon the middle-ground of common sense.

